# A Randomized, Controlled Animal Study: 21% or 100% Oxygen during Cardiopulmonary Resuscitation in Asphyxiated Infant Piglets

**DOI:** 10.3390/children9111601

**Published:** 2022-10-22

**Authors:** Solomon Nyame, Po-Yin Cheung, Tez-Fun Lee, Megan O’Reilly, Georg M. Schmölzer

**Affiliations:** 1 Faculty of Medicine and Dentistry, Monash University, Melbourne, VIC 3000, Australia; 2 Centre for the Studies of Asphyxia and Resuscitation, Neonatal Research Unit, Royal Alexandra Hospital, Edmonton, AB T5H 3V9, Canada; 3 Department of Pediatrics, Faculty of Medicine and Dentistry, University of Alberta, Edmonton, AB T6G 2R, Canada

**Keywords:** infants, resuscitation, chest compressions, asphyxia, oxygen, sustained inflation

## Abstract

Background: During pediatric cardiopulmonary resuscitation (CPR), resuscitation guidelines recommend 100% oxygen (O_2_); however, the most effective O_2_ concentration for infants unknown. Aim: We aimed to determine if 21% O_2_ during CPR with either chest compression (CC) during sustained inflation (SI) (CC + SI) or continuous chest compression with asynchronized ventilation (CCaV) will reduce time to return of spontaneous circulation (ROSC) compared to 100% O_2_ in infant piglets with asphyxia-induced cardiac arrest. Methods: Piglets (20–23 days of age, weighing 6.2–10.2 kg) were anesthetized, intubated, instrumented, and exposed to asphyxia. Cardiac arrest was defined as mean arterial blood pressure < 25 mmHg with bradycardia. After cardiac arrest, piglets were randomized to CC + SI or CCaV with either 21% or 100% O_2_ or the sham. Heart rate, arterial blood pressure, carotid blood flow, and respiratory parameters were continuously recorded. Main results: Baseline parameters, duration, and degree of asphyxiation were not different. Median (interquartile range) time to ROSC was 107 (90–440) and 140 (105–200) s with CC + SI 21% and 100% O_2_, and 600 (50–600) and 600 (95–600) s with CCaV 21% and 100% O_2_ (*p* = 0.27). Overall, six (86%) and six (86%) piglets with CC + SI 21% and 100% O_2_, and three (43%) and three (43%) piglets achieved ROSC with CCaV 21% and 100% O_2_ (*p* = 0.13). Conclusions: In infant piglets resuscitated with CC + SI, time to ROSC reduced and survival improved compared to CCaV. The use of 21% O_2_ had similar time to ROSC, short-term survival, and hemodynamic recovery compared to 100% oxygen. Clinical studies comparing 21% with 100% O_2_ during infant CPR are warranted.

## 1. Introduction

Neonatal, pediatric, and adult resuscitation guidelines recommend 100% oxygen (O_2_) during chest compression [1,2]. Current adult resuscitation guidelines suggest using the highest possible inspired oxygen concentration during cardiopulmonary resuscitation (CPR) (weak recommendation, very-low-certainty evidence) [1,2]. Similarly, pediatric resuscitation guidelines recommend 100% O_2_ during continuous chest compression (CC) with a respiratory rate of 20 to 30/min (CCaV) [3]. In contrast, current neonatal resuscitation guidelines recommend starting with 21% O_2_ for initial respiratory support in term infants in the delivery room and increasing to 100% O_2_ when CC are started [4,5].

Using 100% oxygen during CPR causes hyperoxia, which has been associated with increased mortality in newborn infants (21% O_2_ during CPR associated with reduced relative risk 0.71 [95% CI 0.54 to 0.94], risk difference −0.05 [−0.08 to −0.01], and adults [odds ratio for death of 1.8 (95% CI, 1.5–2.2)] [6,7]. Studies assessing hyperoxia after cardiac arrest in pediatric patients reported no association with in-hospital mortality or poor neurological outcome [8]. Furthermore, hyperoxia leads to generation of oxygen free radicals, which have a role in reperfusion/reoxygenation injury after cardiac arrest [9,10].

During infant CPR, combining CC with a sustained inflation (SI) (CC + SI) significantly reduced median (interquartile range (IQR)) time to ROSC with 248 (41–346) compared to CCaV with 720 (167–720) s (*p* = 0.0292) [11]. Similarly CC + SI significantly achieved ROSC faster compared to the 3:1 compression-to-ventilation ratio in asphyxiated neonatal piglets as well as newborn infants in the delivery room [12,13,14,15,16,17,18]. Furthermore, asphyxiated piglets randomized to either 21% or 100% O2 with 3:1 compression-to-ventilation ratio or CC + SI resulted in a similar time to ROSC [19,20]. Several animal trials examined various oxygen concentrations using adult and neonatal piglets [19,20,21,22,23], while pediatric studies are lacking [24]. Due to the lack of pediatric studies, we aimed to determine if 21% O2 using either CC + SI or CCaV will reduce time to ROSC compared to 100% O2 in infant piglets with asphyxia-induced cardiac arrest. We hypothesized that 21% O2 with either CC + SI or CCaV will reduce time to ROSC compared to 100% O2 in infant piglets requiring CPR after asphyxia-induced cardiac arrest. We also examined differences in hemodynamics and regional perfusion during recovery with different oxygen concentrations.

## 2. Methods

**Figure 1:**
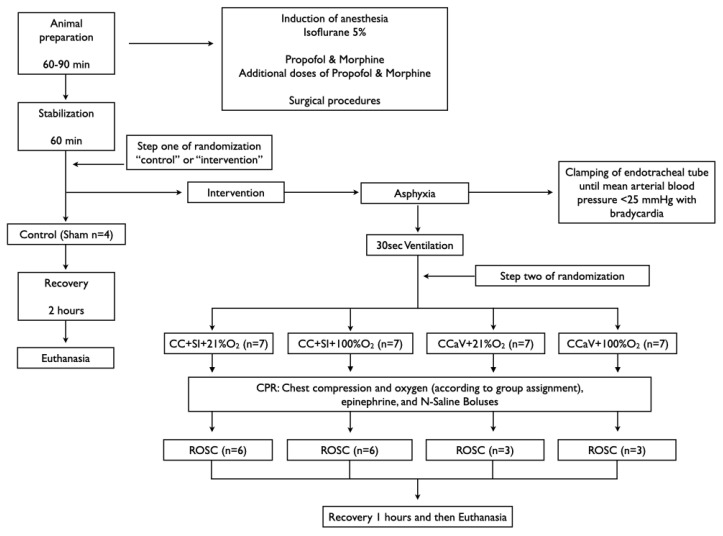
Study flow chart.

Infant mixed breed piglets were obtained on the day of experimentation from the University Swine Research Technology Centre. All experiments were conducted after approval from the Animal Care and Use Committee, University of Alberta (AUP3084), registered at preclinicaltrials.eu (PCTE195), reported according to the ARRIVE guidelines [25]. A graphical display of the study is presented in Figure 1. The authors declare that all supporting data are available within the article.

### 2.1. Inclusion and Exclusion Criteria

Infant piglets aged 20–23 days were included; there were no exclusion criteria.

### 2.2. Randomization

Piglets were randomly allocated to sham, CC + SI or CCaV with 21% or 100% O_2_. Randomization was 1:1 using a computer-generated randomization program (http://www.randomizer.org). A sequentially numbered, sealed, brown envelope containing the allocation “control (sham)” or “intervention” was opened after stabilization (step one of randomization). A second sequentially numbered, sealed, brown envelope was opened just before the commencement of CPR containing the group allocation CC + SI + 21% O_2_, CC + SI + 100% O_2_, CCaV + 21% O_2_, and CCaV + 100% O_2_ (step two of randomization) (Figure 1).

### 2.3. Sample Size and Power Estimates

Our primary outcome measure was the CPR time to achieve ROSC. In a previous study using CCaV + 100% O_2_ a mean (standard deviation-SD) of 700 (120) s of CPR was required to achieve ROSC. A sample size of 14 piglets (7 per group) was sufficient to detect a clinically important (20%) reduction in time to achieve ROSC (i.e., 700 vs. 560 s), with 80% power and a 2-tailed alpha error of 0.05.

### 2.4. Blinding

The person (GMS) assessing cardiac arrest was blinded to group allocation until after cardiac arrest was confirmed. TFL opened the randomization envelope and was solely responsible for setting the inspired oxygen concentration. The remaining team was blinded to the inspired oxygen concentration. However, we were unable to blind the team on resuscitation methods due to the differences in both chest compression techniques. The statistical analysis was blinded to group allocation and only unblinded after the statistical analysis was completed.

### 2.5. Animal Preparation

Following the induction of anesthesia using isoflurane, piglets were intubated via a tracheostomy, and pressure-controlled ventilation (Sechrist infant ventilator, model IV-100; Sechrist Industries, Anaheim, CA, USA) was commenced at a respiratory rate of 16–20 breaths/min and pressure of 20/5 cm H_2_O [11]. Oxygen saturation was kept within 90–100%. Glucose levels and hydration were maintained with an intravenous infusion of 5% dextrose at 10 mL/kg/h [11]. During the experiment, anesthesia was maintained with intravenous propofol 5–10mg/kg/h and morphine 0.1 mg/kg/h [11]. Additional doses of propofol (1–2 mg/kg) and morphine (0.05–0.1 mg/kg) were also given as needed [11]. The piglet’s body temperature was maintained at 38.5–39.5 °C using an overhead warmer and a heating pad [11].

### 2.6. Hemodynamic Parameters

A 5-French Argyle^®^ (Klein-Baker Medical Inc., San Antonio, TX, USA) double-lumen catheter was inserted via the right femoral vein for administration of fluids and medications. A 5-French Argyle^®^ single-lumen catheter was inserted below the right renal artery via the femoral artery for continuous arterial blood pressure monitoring in addition to arterial blood gas measurements. The right common carotid artery was also exposed and encircled with a real-time ultrasonic flow probe (4 mm; Transonic Systems Inc., Ithica, NY, USA) to measure common carotid blood flow [11].

Piglets were placed in a supine position and allowed to recover from surgical instrumentation until baseline hemodynamic measures were stable (minimum of one hour). The ventilator rate was adjusted to keep the partial arterial CO_2_ between 35 and 45 mmHg, as determined by periodic arterial blood gas analysis. Mean systemic arterial pressure, systemic systolic arterial pressure, heart rate, and percutaneous oxygen saturation were continuously measured and recorded throughout the experiment with a Hewlett Packard 78833B monitor (Hewlett Packard Co., Palo Alto, CA, USA) [11].

### 2.7. Respiratory Parameters

A respiratory function monitor (NM3, Respironics, Philips, Andover, MA, USA) was used to continuously measure tidal volume, airway pressures, gas flow, and end-tidal CO_2_. The combined gas flow and end-tidal CO_2_ sensor was placed between the endotracheal tube and the ventilation device [26,27].

### 2.8. Cerebral Perfusion

Cerebral oxygenation was measured using the Invos^TM^ Cerebral/Somatic Oximeter Monitor (Invos 5100, Somanetics Corp., Troy, MI, USA) [28]. The sensors were placed on the right forehead of the piglet and secured with wrap and tape. Light shielding was achieved with a slim cap.

### 2.9. Experimental Protocol

Piglets were randomized into five groups: CC + SI + 21% O_2_, CC + SI + 100% O_2_, CCaV + 21% O_2_, CCaV + 100% O_2_, or control (sham). To reduce selection bias, a two-step randomization process was used. After stabilization, piglets were randomized to “control (sham)” or “intervention” (step one) (Figure 1.). The sham group received the same surgical protocol, stabilization, and equivalent experimental periods without asphyxia. The piglets randomized to “intervention” were exposed to asphyxia, which was achieved by disconnecting the ventilator and clamping the endotracheal tube until cardiac arrest. Cardiac arrest was defined as mean arterial blood pressure < 25 mmHg with bradycardia [11]. After cardiac arrest was confirmed a second envelope containing the assignment “CC + SI + 21% O_2_”, “CC + SI + 100% O_2_”, “CCaV + 21% O_2_”, or “CCaV + 100% O_2_” was opened (step two) (Figure 1). Fifteen seconds after cardiac arrest was confirmed, positive pressure ventilation was performed for 30 s with either a self-inflating bag (Laerdal, Stavanger, Norway) for the CCaV groups or Neopuff T-Piece (Fisher & Paykel, Auckland, New Zealand) for the CC + SI groups with oxygen according to the group allocation. The self-inflating bag was used without a positive end expiratory pressure (PEEP) valve with a gas flow of 10 L/min and a ventilation rate of 20/min. The default settings for the Neopuff T-Piece were a peak inflating pressure (PIP) of 30 cmH_2_O, a PEEP of 5 cmH_2_O, and a gas flow of 10 L/min.

Thirty seconds after positive pressure ventilation, CC was performed on a resuscitation board using the one-hand chest compression technique at a rate of 100/min (guided by a metronome), and operators (G.M.S., P.Y.C.) were switched every two minutes to prevent fatigue [3]. CPR was continued for a maximum time of 10 min. Epinephrine (0.04 mg/kg per dose) was administered intravenously two minutes after the start of positive pressure ventilation, and every minute as needed if no ROSC was observed [3]. Bolus Ringer’s solution (10 mL/min) was given immediately after each epinephrine dose to a total of 10 mL/kg. ROSC was defined as an unassisted heart rate ≥ 100/min for 15 s After ROSC, piglets were monitored for 30 min. At the end of experimentation, piglets were euthanized with an intravenous overdose of sodium pentobarbital (100 mg/kg).

After euthanizing, the left ventricle and right basal part of the lung lobe were snap frozen in liquid nitrogen and stored in −80 °C until subsequent analysis. The brain was removed from the skull and placed in ice-cold 2-methylbutane for 10 min before storing at –80 °C. Only tissue samples from piglets surviving 30 min after the intervention were collected (sham-operated n = 4; CC + SI + 21% O_2_ n = 5; CC + SI + 100% O_2_ n = 6, CCaV + 21% O_2_ n = 3, and CCaV + 100% O_2_ n = 3).

Tissue samples were homogenized in a lysis buffer (0.5% Tween-20/PBS containing a protease inhibitor cocktail). Homogenized samples were centrifuged at 3000×g for 10 min at 4 °C. The supernatants were retained, and protein concentration was quantified using the Bradford method.

Levels of lactate and glutathione (GSH) in myocardial tissue, lung tissue, and frontoparietal cortex homogenates were used as surrogate markers for hypoxic damage and oxidative stress; values were expressed relative to protein. Lactate was measured using an enzyme assay for absorbance of NADH at 340 nm. Tissues for the lactate assay were prepared by homogenization in 6% perchloric acid with 0.5 mM EGTA and then neutralized after separation with 5 M potassium carbonate. Both total GSH and oxidized glutathione (GSSG) were measured using a commercially available assay kit (#703002, Cayman Chemical, Ann Arbor, MI, USA). GSH and GSSG were then assayed according to manufacturer instructions, with GSSG assayed by derivatizing GSH in the sample preparations with 2-vinylpyridine solution (Cat. #13,229–2, Aldrich Chemical Company, Milwaukee, WI, USA). All assay absorbance readings were measured using a Molecular Devices Spectramax 190 Absorbance Microplate Reader (Molecular Devices, LLC. Sunnyvale, California, CA, USA) [19,20].

## 3. Data Collection and Analysis

Demographics of study piglets were recorded. Transonic flow probes, heart rate, and pressure transducer outputs were digitized and recorded with LabChart^®^ programming software (ADInstruments, Houston, TX, USA). Airway pressures, gas flow, V_T_, and ETCO_2_ were measured and analyzed using Flow Tool Physiologic Waveform Viewer (Philips Healthcare, Wallingford, CT, USA).

The data are presented as mean (standard deviation–SD) for normally distributed continuous variables and median (interquartile range-IQR) when the distribution was skewed. For all respiratory parameters, continuous values during CPR were analyzed. The data was tested for normality and compared using either one-way (e.g., time to ROSC) or two-way (e.g., hemodynamic changes) ANOVA. Fisher exact test was used for proportion comparisons. *p*-values are 2-sided, and *p* < 0.05 was considered statistically significant. Statistical analyses were performed with SigmaPlot (Systat Software Inc., San Jose, CA, USA).

## 4. Results

Thirty-two infant piglets, 20–23 days old (weight from 7.0–9.3 kg), were randomly assigned to the CC + SI + 21% O_2_, CC + SI + 100% O_2_, CCaV + 21% O_2_, CCaV + 100% O_2_, or sham groups. There were no differences in baseline parameters between groups (Table 1).

### 4.1. Resuscitation and Primary Outcome

Asphyxia time and degree of asphyxiation were not different between groups (Table 2). In the CCaV group, median (IQR) time to ROSC was 600 (50–600) and 600 (95–600) s with 21% and 100% O_2_ (Table 2). With CCaV + 21% O_2_ and CCaV + 100% O_2_ 3 (43%), piglets in each group achieved ROSC (Table 2).

In the CC + SI group, time to ROSC was 107 (90–440) and 140 (105–200) s with 21% and 100% O_2_ (Table 2). With CC + SI + 21% O_2_ and CC + SI + 100% O_2_ 6 (86%), piglets achieved ROSC (Table 2). The number of epinephrine doses received between 21% and 100% O_2_, was not significantly different; however, piglets randomized to CC + SI received less epinephrine. There was no difference in blood gases before and after resuscitation except at 30 min after ROSC compared to sham (Table 3).

### 4.2. Respiratory and Hemodynamic Parameters

**Figure 2:**
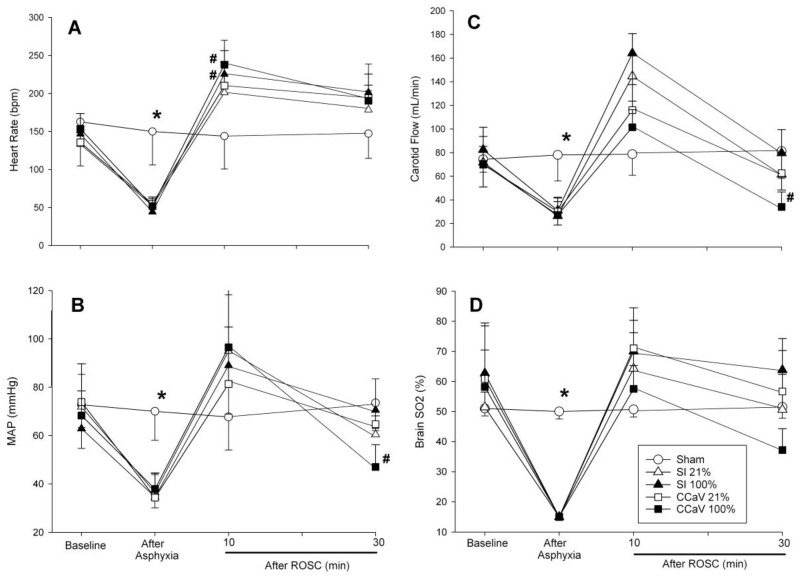
Changes in heart rate (**A**), mean arterial blood pressure (**B**), carotid blood flow (**C**), and cerebral oxygenation (**D**) with 21% and 100% O_2_ during continuous chest compression during sustained inflation (CC + SI), continuous chest compression with asynchronized ventilation (CCaV). * Significantly different from all intervention groups, # significantly different from sham-operated group at the same time point.

Tidal volume, gas flow, and airway pressures are presented in Table 4. Overall, minute ventilation and positive end expiratory pressure was significantly improved with CC + SI compared to CCaV (Table 4). There were no differences in changes in cerebral oxygenation, mean arterial blood pressure, heart rate, and carotid blood flow during baseline, asphyxia, CPR, and recovery (Figure 2).

### 4.3. Injury Markers

We identified no difference in GSH, GSSG, and GSSG/GSH ratios in the lung or myocardial tissues or in the frontoparietal cortex between 21% and 100% O_2_ with CC + SI or CCaV (Table 5). The pattern in the differences in tissue lactate was not consistent.

## 5. Discussion

Current adult, pediatric, and neonatal resuscitation guidelines recommend 100% O_2_ during CPR [1]. Currently, there are no human data in neonatal or pediatric patients [21,24,29], which forces the guidelines to solely rely on extrapolation from adult studies [22,23]. Indeed, a recent pediatric animal study compared 21% with 100% O_2_ during CPR and reported similar rates of survival; however, piglets exposed to 100% oxygen had cerebral hyperoxia during resuscitation, increased mitochondrial-derived reactive oxygen species, and oxidative injury following cardiac arrest [24]. In the current study, we randomized piglets to CC + SI or CCaV with either 21% or 100% O_2_; the results of our study can be summarized as follows: (1) CC + SI reduced time to ROSC compared to CCaV Table 2); (2) time to ROSC was not different irrespective of oxygen concertation (Table 2); (3) minute ventilation was significantly increased with CC + SI (Table 4); and (4) alveolar oxygen delivery was significantly decreased with 21% O_2_, while the amount of oxidative cell injury was similar between groups (Table 5).

Neonatal resuscitation studies comparing 21% with 100% O_2_ during mask ventilation of term infants reported that mortality was reduced in infants resuscitated with air (relative risk, 0.71 [95% CI 0.54–0.94]; risk difference, −0.05 [−0.08 to–0.01]) [6]. Similarly, a cohort study in 6326 adult patients receiving CPR with 100% O_2_ reported hyperoxia in 1156 (18%), hypoxia in 3999 (63%), and normoxia in 1171 (19%) [7]. The hyperoxia group had significantly higher in-hospital mortality (732/1156 [63%; 95% CI, 60–66%]) compared to either normoxia or hypoxia with an odds ratio for death of 1.8 (95% CI, 1.5–2.2) [7]. This is further supported by animal studies in adults and newborns, which both reported higher mortality in animals resuscitated with 100% O_2_ [19,20,21,22,23,30]. In the current study, we also observed that 100% O_2_ had a lower likelihood of ROSC compared to 21% O_2_; however, once ROSC was achieved, mortality rates were similar between both oxygen concentrations. Furthermore, oxidative stress injury markers were similar between 21% and 100% O_2_ (Table 5). In comparison, Marquez et al. compared 21% with 100% O_2_ during pediatric CPR and reported cerebral hyperoxia during CPR and immediately following ROSC as well as higher cerebral mitochondrial reactive oxygen species production in piglets resuscitated with 100% compared to 21% O_2_ [24]. Neither the study by Marquez et al. nor the current study examined longer-term neurological outcomes, which are a necessity before clinical trials could be performed.

We have previously reported that CC + SI reduces time to ROSC by up to 75% during neonatal and infant CPR of asphyxiated piglets and newborn infants [11,12,13,15]. In the current study, time to ROSC was reduced with CC + SI compared to CCaV irrespective of percentage of oxygen used (107 s, 140 s, 600 s, 600 s with CC + SI + 21% or 100% O_2_ and CCaV + 21% or 100% O_2_, respectively). Time to ROSC was 77–82% shorter with CC + SI, whether using 21% or 100% O_2_, compared to the CCaV technique (Table 2). Furthermore, the number of piglets achieving ROSC doubled from 43% with CCaV to 86% with CC + SI (Table 2). Under CC + SI, there was an additional 24% reduction in time to ROSC using 21% O_2_ compared to 100% oxygen (Table 2). The results demonstrate that the CC + SI technique while providing 21% O_2_ might be the most effective at achieving ROSC in asphyxiated infant piglets. Our results suggest that CC + SI with 21% O_2_ might be the most effective approach during infant CPR.

Cardiac arrest in infants and children does not usually result from a primary cardiac cause and rather is the terminal result of progressive respiratory failure [31,32]; therefore, adequate alveolar oxygen delivery might facilitate ROSC quicker. Similarly, neonatal cardiac arrest is caused by hypoxia/asphyxia with the main resuscitation focus on providing adequate ventilation [1]. The current pediatric resuscitation guidelines recommended ventilation rates of 12–20/min during pediatric CPR [31,32], while the neonatal guidelines recommended ventilation rates of 30/min [1]. Sutton et al. compared ventilation rates of 10/min to 25–30/min, depending on the age of pediatric patient, in pediatric patients needing CPR within the Collaborative Pediatric Critical Care Research Network [33]. High ventilation rates were associated with a higher odds of survival to discharge (odds ratio, 4.73; *p* = 0.029), even after controlling for location of CPR, initial rhythm, and time of day [33]. Using higher ventilation rates might be beneficial in children as they have higher baseline ventilation rates and their cardiac arrests are more likely due to respiratory deterioration and therefore restore adequate oxygenation and ventilation during CPR more quickly. In the current study, CC + SI had a 6 times higher minute ventilation (Table 4), which might have been a contributing factor for a faster time to ROSC. Unfortunately, no blood gas at the time of ROSC was collected to assess if CC + SI would have resulted in hypocarbia, which is a limitation of the current study.

## 6. Limitations

Our piglet asphyxia model closely mimics asphyxia events in children, leading to bradycardia with cardiac arrest, compared to cardiac arrest induced by ventricular fibrillation [11]. However, several limitations should be considered: All piglets were sedated/anesthetized and intubated with a tightly sealed endotracheal tube to prevent any endotracheal tube leak, which may not occur in all infant patients [11]. However, our results are still clinically relevant as the distribution of cardiac output during asphyxia episodes are qualitatively similar. As we administered the first dose of epinephrine 90 s after CC was initiated and then every 60 s, we have slightly deviated from the currently recommended resuscitation guidelines, which may have influenced our results [3].

## 7. Conclusions

CC + SI reduced time to ROSC and improved survival compared to CCaV, and 21% O_2_ had similar time to ROSC, short-term survival, and hemodynamic recovery compared to 100% oxygen. This might be of clinical importance as there is the potential to improve outcomes in infants with cardiac arrest; however, further studies are warranted.

## Figures and Tables

**Table 1:** Baseline characteristics.

	Sham(n = 4)	CC + SI		CCaV		*p*-Value
		21% Oxygen (n = 7)	100% Oxygen (n = 7)	21% Oxygen (n = 7)	100% Oxygen (n = 7)	
Age (days)	22 (21–22)	22 (21–23)	22 (21–22)	23 (21–25)	22 (21–24)	0.286
Weight (kg)	8.0 (7.4–8.6)	7.9 (7.7–8.1)	7.9 (7.0–8.6)	7.8 (7.5–9.1)	8.1 (6.9–9.3)	0.889
Sex (male/female)	1/3	4/3	4/3	6/1	2/5	0.196
Heart rate (bpm)	147 (117–224)	134 (127–145)	144 (139–158)	132 (128–148)	154 (136–173)	0.235
MAP (mmHg)	73 (64–82)	68 (62–77)	61 (55–72)	70 (61–88)	63 (60–65)	0.198
Carotid flow (mL/min)	73 (62–96)	71 (52–85)	77 (66–129)	74 (55–82)	66 (46–85)	0.574
Cerebral oxygenation (%)	52 (49–53)	51 (46–57)	67 (45–77)	57 (53–67)	60 (44–75)	0.489
pH	7.39 (7.33–7.46)	7.42 (7.35–7.46)	7.46 (7.42–7.49)	7.47 (7.41–7.48)	7.44 (7.40–7.49)	0.351
Base excess (mmol/L)	2 (−3.3~4.3)	0 (−2.0~4.0)	2 (0~3.0)	2 (1.0~3.1)	2 (1.0~3.0)	0.649
paCO_2_ (torr)	39 (32–40)	39 (36–42)	37 (36–38)	36 (35–39)	38 (36–42)	0.942
SaO_2_ (%)	97 (97–99)	98 (96–98)	98 (94–99)	99 (99–99)	96 (94–99)	0.181
Lactate (mmol/L)	2.1 (1.6–4.8)	2.1 (1.8–2.9)	2.4 (2.2–2.9)	2.1 (1.7–3.3)	2.4 (1.8–2.5)	0.878

Data are presented as median (IQR); MAP—Mean arterial blood pressure, CC + SI—continuous chest compression during sustained inflation, CCaV—continuous chest compression with asynchronized ventilation.

**Table 2:** Characteristics of asphyxia, resuscitation, and survival of asphyxiated piglets.

		CC + SI		CCaV		*p*-Value
		21% Oxygen (n = 7)	100% Oxygen (n = 7)	21% Oxygen (n = 7)	100% Oxygen (n = 7)	
Asphyxia time (s) ^†^		330 (305–451)	350 (324–429)	315 (263–435)	335 (240–435)	0.91
Immediately before resuscitation	pH^†^	6.94 (6.93–7.07)	7.02 (6.89–7.12)	7.09 (6.94–7.14)	7.04 (6.95–7.13)	0.46
	paCO_2_ (torr) ^†^	107 (83–114)	88 (74–103)	89 (80–110)	94 (84–105)	0.52
	Lactate (mmol/L) ^†^	9.7 (8.8–10.4)	9.0 (7.4–11.9)	8.2 (6.3–9.1)	7.6 (6.5–9.5)	0.24
	Base excess (mmol/L)	−8 (−10~−6)	−8 (-11~−5)	−6 (−9~−2)	−3 (−9~−2)	0.19
Resuscitation	Received epinephrine (n)	6 (86%)	7 (100%)	5 (71%)	7 (100%)	0.25
	Epinephrine doses (n) ^†^	1 (1–7)	2 (1–3)	9 (0–9)	9 (1–9)	0.33
	Ringer solution bolus (mL) ^†^	10 (10–80)	20 (5–25)	75 (0–90)	70 (5–90)	0.37
Achieving ROSC		6 (86%)	6 (86%)	3 (43%)	3 (43%)	0.13
ROSC time (s) ^†^		107 (90–440)	140 (105–200)	600 (50–600)	600 (95–600)	0.27
Survival after ROSC		5 (83%)	6 (100%)	3 (100%)	3 (100%)	0.55

Data are presented as n (%), unless indicated ^†^ median (IQR), ROSC—return of spontaneous circulation, CC + SI—continuous chest compression during sustained inflation, CCaV—continuous chest compression with asynchronized ventilation.

**Table 3:** Blood gas changes before and after resuscitation.

	Sham	CC + SI		CCaV		*p*-Value
		21% Oxygen	100% Oxygen	21% Oxygen	100% Oxygen	
pH						
Baseline	7.39 (7.33–7.46)	7.42 (7.35–7.46)	7.46 (7.42–7.49)	7.47 (7.41–7.48)	7.44 (7.40–7.49)	0.351
After asphyxiation	N/A	6.94 (6.93–7.07) ^#^	7.02 (6.89–7.12) ^#^	7.09 (6.94–7.14) ^#^	7.04 (6.96–7.13) ^#^	0.460
30 min after resuscitation	7.42 (7.38–7.463)	7.35 (7.26–7.40)	7.29 (7.17–7.34)	7.32 (7.19–7.36)	7.38 (7.24–7.46)	0.178
PaCO_2_ (torr)						
Baseline	39 (32–40)	39 (36–42)	37 (36–38)	36 (35–39)	38 (36–42)	0.942
After asphyxiation	N/A	106 (83–114) ^#^	88 (74–103) ^#^	89 (80–110) ^#^	94 (84–105) ^#^	0.516
30 min after resuscitation	36 (34–40)	39 (29–44)	40 (38–56)	37 (36–52)	37 (31–42)	0.456
Base excess (mmol/L)						
Baseline	2 (−3~4)	0 (−2~4)	2 (0~3)	2 (1~ 3)	2 (1~3)	0.649
After asphyxiation	N/A	−8 (−10~−6) ^#^	−8 (−11~−5) ^#^	−6 (−9~ −2) ^#^	−5 (−9~−3) ^#^	0.335
30 min after resuscitation	1 (−4~3)	−6 (−9~−4) * ^#^	−7 (−11~−6) * ^#^	−7 (−8~ −5) * ^#^	−9 (−10~−4) * ^#^	0.033
Lactate (mmol/L)						
Baseline	2.1 (1.6–4.8)	2.1 (1.8–2.9)	2.4 (2.2–2.9)	2.1 (1.7–3.3)	2.4 (1.8–2.5)	0.878
After asphyxiation	N/A	9.7 (8.8–10.5) ^#^	9.0 (7.4–11.9) ^#^	8.2 (6.3–9.1) ^#^	7.6 (6.5–9.5) ^#^	0.235
30 min after resuscitation	2.4 (1.8–4.8)	6.9 (5.6–8.6) * ^#^	8.2 (6.7–10.2) * ^#^	7.3 (6.5–8.6) * ^#^	7.0 (6.8–8.7) * ^#^	0.007

Data are presented as median (IQR); N/A—not applicable; CC + SI—continuous chest compression during sustained inflation, CCaV—continuous chest compression with asynchronized ventilation; * *p* < 0.05 vs. sham, ^#^ *p* < 0.05 vs. respective baseline values.

**Table 4:** Respiratory Parameters during the duration of Chest Compression.

Respiratory Parameter	CC + SI + 21% O_2_ (n = 7)	CC + SI + 100% O_2_ (n = 7)	CCaV + 21% O_2_ (n = 7)	CCaV + 100% O_2_ (n = 7)	*p*-Value
Positive End Expiratory Pressure (cmH_2_O)	31.6 (4.3) *	27.3 (3.1) *	6.8 (0.6)	7.2 (1.1)	<0.001
Peak Inflation Flow (mL/min)	7.4 (1.2)	6.8 (1.1)	3.7 (1.2)	4.2 (-1.5)	0.794
Peak Expiratory Flow (mL/min)	−15.5 (3.4)	−13.4 (2.0)	−8.0 (3.5)	−7.3 (3.8)	0.618
Tidal Volume (mL/kg) ^#^	25.4 (6.2)	22.4 (6.9)	19.8 (8.2)	18.8 (9.9)	0.675
Minute Ventilation (mL/min) ^#^	2678 (664) *	2418 (591) *	401 (172)	530 (436)	0.003

Data are presented as mean (standard deviation), unless indicated # median (interquartile range); * *p* < 0.05 between CC + SI and CCaV. CC + SI—chest compression (CC) during sustained inflation (SI), CCaV—continuous chest compression with asynchronized ventilation, O_2_—oxygen.

**Table 5:** Injury markers.

	Sham	CC + SI		CCaV		*p*-Value
	(n = 4)	21% Oxygen (n = 5)	100% Oxygen (n = 6)	21% Oxygen (n = 3)	100% Oxygen (n = 3)	
Left ventricle						
GSSG (µM/mg protein)	171 (28)	191 (54)	248 (123)	177 (49)	177 (52)	0.53
GSH (µM/mg protein)	879 (158)	805 (158)	1029 (394)	1130 (311)	662 (60)	0.44
GSSG/GSH	0.19 (0.05)	0.24 (0.07)	0.26 (0.1)	0.17 (0.03)	0.27 (0.08)	0.32
Lactate (µM/mg protein)	0.11 (0.05)	0.33 (0.15)*	0.28 (0.06)	0.23 (0.04)	0.29 (0.06)	0.015
Lung						
GSSG (µM/mg protein)	138 (32)	185 (59)	198 (51)	169 (46)	201 (31)	0.47
GSH (µM/mg protein)	909 (121)	883 (155)	802 (314)	712 (173)	583 (197)	0.49
GSSG/GSH	0.15 (0.04)	0.21 (0.09)	0.28 (0.1)	0.25 (0.09)	0.41 (0.21)	0.07
Lactate (µM/mg protein)	0.15 (0.02)	0.25 (0.03)	0.27 (0.03)*	0.25 (0.03)	0.34 (0.3) *	0.004
Brain						
GSSG (µM/mg protein)	129 (31)	119 (41)	197 (48)	117 (28)	106 (22)	0.07
GSH (µM/mg protein)	797 (226)	638 (205)	606 (235)	633 (307)	438 (213)	0.55
GSSG/GSH	0.19 (0.05)	0.22 (0.12)	0.35 (0.13)	0.25 (0.17)	0.34 (0.21)	0.39
Lactate (µM/mg protein)	0.12 (0.02)	0.25 (0.05) *	0.22 (0.01)	0.23 (0.04)	0.29 (0.13) *	0.010

Data are presented as mean (SD); CC + SI—continuous chest compression during sustained inflation, CCaV—continuous chest compression with asynchronized ventilation; * *p* < 0.05 vs. sham (Tukey).

## Data Availability

Not applicable.

## A 140-character Tweet

The results of 21% oxygen during infant CPR showed similar outcomes to those of 100% oxygen; clinical trials are warranted.

## Abbreviations

CC	Chest compression
CPR	Cardiopulmonary resuscitation
ROSC	Return of spontaneous circulation
SI	Sustained Inflation
O_2_	Oxygen
PPV	Positive pressure ventilation
3:1	Compression to Ventilation ratio
IQR	Interquartile range
